# Characterization of the Interaction of Polymeric Micelles with siRNA: A Combined Experimental and Molecular Dynamics Study

**DOI:** 10.3390/polym14204409

**Published:** 2022-10-19

**Authors:** Franck Marquet, Filip Stojceski, Gianvito Grasso, Viorica Patrulea, Andrea Danani, Gerrit Borchard

**Affiliations:** 1 Section of Pharmaceutical Sciences, University of Geneva, CMU—Rue Michel-Servet 1, 1211 Geneva, Switzerland; 2 Institute of Pharmaceutical Sciences of Western Switzerland (ISPSO), University of Geneva, CMU—Rue Michel-Servet 1, 1211 Geneva, Switzerland; 3 Dalle Molle Institute for Artificial Intelligence (IDSIA), University of Italian Switzerland (USI), University of Applied Science and Art of Southern Switzerland (SUPSI), Polo Universitario Lugano—Campus Est, Via la Santa 1, CH-6962 Lugano-Viganello, Switzerland; 4 Institute of Biomedical Engineering, Department of Engineering Science, University of Oxford, Oxford OX3 7DQ, UK

**Keywords:** polymeric micelles, isothermal titration calorimetry, molecular dynamics, capillary zone electrophoresis, binding affinity, complexation efficiency

## Abstract

The simulation of large molecular systems remains a daunting challenge, which justifies the exploration of novel methodologies to keep computers as an ideal companion tool for everyday laboratory work. Whole micelles, bigger than 20 nm in size, formed by the self-assembly of hundreds of copolymers containing more than 50 repeating units, have until now rarely been simulated, due to a lack of computational power. Therefore, a flexible amphiphilic triblock copolymer (mPEG_45_-α-PLL_10_-PLA_25_) containing a total of 80 repeating units, has been emulated and synthesized to embody compactified nanoconstructs of over 900 assembled copolymers, sized between 80 and 100 nm, for siRNA complexing purposes. In this study, the tailored triblock copolymers containing a controlled number of amino groups, were used as a support model to address the binding behavior of STAT3-siRNA, in the formation of micelleplexes. Since increasingly complex drug delivery systems require an ever more optimized physicochemical characterization, a converging description has been implemented by a combination of experimentation and computational simulations. The computational data were advantageous in allowing for the assumption of an optimal N/P ratio favoring both conformational rigidifications of STAT3-siRNA with low competitive phenomena at the binding sites of the micellar carriers. These calculations were consistent with the experimental data showing that an N/P ratio of 1.5 resulted in a sufficient amount of complexed STAT3-siRNA with an electrical potential at the slipping plane of the nanopharmaceuticals, close to the charge neutralization.

## 1. Introduction

The use of siRNA is an effective approach to treating various pathologies such as rare genetic disorders, cardiovascular, viral or cancerous diseases [1,2,3]. The delivery of siRNA is mostly accomplished *via* the use of cationic transfection agents [4,5]. Cationic polymers have garnered tremendous interest due to their ability to protect siRNAs and favor cellular uptake [6]. A large selection of amine-based polycations can be used including the iconic poly-*L*-lysine (PLL). Unfortunately, the clinical use of synthetic polymers is hindered by too low transfection efficiency and/or high toxicity [7].

With respect to polyplexes and/or micelleplexes, the most common stoichiometric determination is based on the N/P charge ratio or more precisely, on the ratio of positive charges of amino groups (N) to negative charges of phosphate groups (P) [8]. Usually, this ratio is greater than unity, which results in positive zeta-potential (ZP) values [9] that ensure a high loading capacity and an improved transfection capability. Although optimal complexation does not necessarily mean optimal transfection, complex stability remains a prerequisite to be observed through optimal loading at charge neutralization ratio. Indeed, the safety concerns related to high cytotoxicity and instability remain the main bottleneck limiting clinical translation.

To overcome such drawbacks, efforts have been made to obtain biocompatible polymeric architectures, serving as siRNA supports while presenting non-toxic and biodegradable features. To this end, triblock copolymeric micelles consisting of molecularly well-defined, highly pure and non-cytotoxic entities have been developed [10]. The controlled character of an optimal charge length of 10 lysines, conceptualized first by a computational method [11], helps evaluate both experimental and computational complexation efficacies. As a result, a methoxy poly(ethylene glycol)-*block*-poly(α-*L*-lysine)-*block*-poly(*D*,*L*-lactic acid) (mPEG-α-PLL-PLA) triblock copolymer has been elegantly exploited as a micellar scaffold. The enthalpy profile of the micelleplex formation was systematically examined.

On the experimental side, the complexation behavior was characterized by different methods including capillary zone electrophoresis (CZE) and isothermal titration calorimetry (ITC). Thereby, complexation efficacy and key parameters pertaining to the macroscopic dissociation process, as described by an apparent dissociation constant (K_d_), were conspicuously delineated and used to estimate binding affinity.

On the computational side, the molecular geometry of triblock copolymeric branches binding to STAT3-siRNA was simulated by molecular dynamics (MD). The goal was to artificially emulate a micelleplex containing fewer polymeric chains to support experimental results.

In this regard, a coupled approach based on analytical methods together with computational modeling may provide fruitful information on the molecular mechanisms that drive the complex formation of carrier-payload systems, such as polymer-siRNA complexes. This aims to better define an N/P ratio closest to the charge neutralization point, with optimal complexation of the payload.

## 2. Materials and Methods

### 2.1. Materials

Human STAT3-siRNA (1385 atoms and 13,330.21 Da) of the following sequence was provided by Dharmacon Inc. (Horizon Discovery Ltd., Waterbeach, UK): (sense strand: 5’-GGAGCAGCACCUUCAGGAUdTdT-3’; antisense strand: 5’-AUCCUGAAGGUGCUGCUCCdTdT-3’). GeneRuler Ultra Low Range DNA Ladder containing TriTrac DNA Loading Dye (6X) was purchased from Thermo Fisher Scientific (Vilnius, Lithuania). Agarose, Quant-iT^TM^ RiboGreen^®^ RNA Reagent and Kit, SYBR Safe DNA gel stain, UltraPure^TM^ TBE Buffer, 10X and UltraPure^TM^ DNase/RNase-Free Distilled Water were provided by Invitrogen (Thermo Fischer Sci., Carlsbad, CA, USA). Methanol (HPLC grade MeOH), ≥99.8% was provided by Fischer Scientific (Thermo Fischer Sci., Loughborough, UK). Sodium chloride (NaCl) for the analysis was obtained from Applichem GmbH (Darmstadt, Germany). 4-Methylphtalalic anhydride 96%, phosphoric acid, extra pure, 85 wt% solution in water, sodium hydroxide pellets, 98.5%, were purchased from Acros Organics (Geel, Belgium). Hydrochloric acid fuming ≥ 37% (HCl), HEPES (N-[2-Hydroxyethyl)piperazine-N’-[2-ethanesulfonic acid]) sodium salt, poly(ethylene glycol) average Mn 6000 (PEG), Trizma^®^ hydrochloride (TRIS HCl) and Tween 20 were obtained from Sigma Aldrich (St-Louis, MO, USA).

### 2.2. Preparation of siRNA-Loaded Micelleplexes

An mPEG-α-PLL-PLA triblock copolymer was synthesized and the polymeric micelles were prepared, according to a procedure recently described [8]. Briefly, polymeric micelles were prepared using a solvent evaporation technique at a stock concentration of 2 mg/mL. The resulting micelles were filtered through a 0.22 μm pore size hydrophilic PVDF membrane and diluted in Milli-Q water at the corresponding charge ratio. A stock standard solution of STAT3-siRNA was prepared at 100 μM in RNase-free water and stored at 253 K (−20 °C) until use. Standard solutions were freshly prepared by diluting the stock solution to the appropriate concentrations and verified using a Nanodrop ND 1000 UV/Vis Spectrophotometer (NanoDrop Technologies, Wilmington, DE, USA), each at 260 nm wavelength, before use. STAT3-siRNA was complexed with the cationic micelles in RNase-free water at different N/P charge ratios, vortexed during 10 s and left at room temperature for at least 20 min before analysis.

### 2.3. Characterization of Size and Zeta Potential Measurements

Micelleplexes with different N/P ratios, each containing 1 μM siRNA, were prepared for the measurement of hydrodynamic diameters. Determination of zeta potential values was established for the micelleplexes with different N/P ratios containing 5 μM siRNA diluted in 1 mM NaCl. Samples were prepared at different N/P ratios. The measurements were carried out using a Zeta-sizer Nano (Malvern, Worcestershire, UK) at 298 K. The results for the size and the zeta potential were compared to those obtained by nanoparticle tracking analysis (NTA) using a PMX-220 TWIN ZetaView (Particle Metrix GmbH, Inning am Ammersee, Germany). Micelleplexes were prepared at different ratios, containing 1 μM siRNA and diluted 10 times before measurement. The measurements were performed in a pulse-sensed electric field. The morphology of the dried micelleplexes was imaged using a JSM-8001FA scanning electron microscope (SEM, JEOL, Tokyo, Japan) at an acceleration voltage of 5 kV after the coating samples with a 20 nm gold layer and compared with transmission electron microscopy (TEM), at a high voltage of 80 kV, using a Tecnai G2 12 (FEI Company, Eindhoven, The Netherlands). All samples were prepared to contain 1 μM siRNA and diluted to obtain 0.04 mg/mL of polymeric materials of each ratio. Three to 5 μL of the samples were spotted on appropriate supports and dried at room temperature over 48 h before imaging.

### 2.4. Gel Retardation Assay

Polymeric micelles were allowed to form complexes containing 100 ng/μL STAT3-siRNA at different N/P ratios. The quality of complexation was determined by gel electrophoresis. The complexes (10 μL) were mixed and allowed for complex formation at room temperature for 10 min before adding the loading dye (2 μL). The samples were then loaded onto a 4% gel agarose in a TBE buffer containing a SYBR Safe migration dye for detection of non-complexed oligonucleotides. The electrophoresis was performed at 80 V for 50 min in a horizontal electrophoresis apparatus and visualized by exposure to UV-illumination (302 nm) with a molecular imaging Gel Dox XR system (Bio-Rad Laboratories, Inc. Hercules, CA, USA).

### 2.5. Capillary Zone Electrophoresis (CZE)

#### 2.5.1. Instrumentation

Electrophoretic data were generated using an HP G1600AX 3D CE system, (Agilent Technologies, Waldbronn, Germany) equipped with a diode-array detector. Separations were carried out in an uncoated fused silica capillary (Composite, Metal Service, Worcestershire, UK) with a 50 μm inner diameter and 48.5 cm total length, thermostatted at 298 K. A suitable optical window was conceived allowing for an alignment interface to the UV detector. UV detection was set at 210 and 260 nm, with a bandwidth of 10 nm. Prior to its first use, the capillary was sequentially washed (5 bar) with HPLC grade MeOH, 1M HCl, 1 M NaOH, Milli-Q H_2_O (1 min each). The background electrolyte (BGE) was constituted with 100 mM Tris HCl, 100 mM HEPES Na+, 100 mM NaCl, 0.1% PEG 6000, 0.1% Tween 20 and adjusted to pH 7.4 with 0.1 M H_3_PO_4_. The capillary was rinsed (1 bar) with BGE (5 min) and the separation voltage (10 kV) was applied for 5 min, driving a current flow of 52 μA at a power of 0.5 W. Prior to each sample injection, the capillary was successively washed with water (3 min) and then equilibrated with BGE (3 min). Samples were kept at ambient temperature in an autosampler and hydrodynamically injected by applying a pressure of −50 mbar during 7 s to overcome sensitivity issues at high N/P ratios. The separated BGEs were refreshed every ten runs to keep a low current difference (0.32 μA ± 0.08). Following each working day, the capillary was washed with water (5 min) and flushed with air (5 min) for dry storage. PEG present in the BGE served as a dynamic coating, allowing a reduction of the electro-osmotic flow for the detection of siRNAs using a short-end injection technique. The effective capillary length (L_e_) was reduced by performing the injection on the detector side (L_e_ = 8.5 cm) instead of on the conventional injection on its opposite side (L_e_ = 40 cm), to detect siRNAs in less than 5 min. This method was adapted by using a silica capillary from a previous publication by Furst et al. [12]. Basic conditioning allows the hydrolysis of siloxanes. The addition of 0.1% Tween 20 was used to decrease the adsorption of siRNA to the capillary wall.

#### 2.5.2. Standard Solutions and Samples

4-Methylphtalate anhydride (4 μg/mL) was added to all final concentrations of the siRNA standard solutions and samples, as an internal standard. As previously mentioned for quantification [12], the areas under the curve (AUC) at 260 nm at the migration time of siRNA were normalized to the AUC at 210 nm at the migration time of 4-methylphthalic anhydride in accordance with the following Equation (1):

(1) Normalized peak ratios=A260T260A210T210,

A calibration curve was established using nine concentration levels of siRNA (k = 9) in triplicate (n = 3) between 0.5 to 8 μM prepared in RNase-free water with a dispersion around a linear relationship (r^2^ = 0.9955). The micelleplexes were prepared to contain a 7 μM siRNA concentration at different N/P ratios, ranging from 0 to 5. Finally, the percentage of complexed siRNA was calculated by subtraction of non-complexed siRNA normalized to siRNA alone, used as control.

### 2.6. RiboGreen^®^ Fluorescence-Based Assay

The concentration of non-complexed siRNA was determined using a Quant-iT RiboGreen RNA assay (Invitrogen, Life Technologies, Eugene, OR, USA), following the manufacturer’s protocol. Micelleplexes were prepared at various N/P ratios with 37.5 nM siRNA concentrations and transferred onto a 96-well plate. The fluorescence was measured using a Synergy Mx microplate reader (BioTeK, Winooski, VT, USA) with standard excitation and emission wavelengths set at 485 ± 10 nm and 530 ± 12.5 nm, respectively.

### 2.7. Molecular Dynamics Simulations

The human STAT3-siRNA starting coordinates were built in the canonical B-form using the AMBER NAB tool. The mPEG-α-PLL-PLA triblock copolymer was modeled using the Avogadro chemical editor [13]. In particular, the initial conformation of the PLL moiety was obtained from the PEPFOLD-3 server [14]. Partial charges of the triblock copolymer were obtained using the AM1-BCC method [15], widely used in the field of polymer partial charge calculation [11,16,17]. The general amber force field (GAFF) [18] was chosen to describe the siRNA and the triblock copolymer molecules. The AMBER99-ILDN force field [19] was chosen to describe the siRNA and the triblock copolymer topologies. The triblock copolymer and the siRNA were set in the center of the cubic box with a 10 nm side and a minimum starting distance of 1.5 nm between them. The system was subsequently solvated with TIP3P water [20] and filled with ions (Cl^−^ and Na^+^) at a concentration of 0.15 M. The GROMACS 2020.1 [21,22] package was adopted for performing molecular dynamics (MD) simulations. A 100 ps position restrain MD was carried out in respect of the NVT ensemble using the v-rescale [23] thermostatted at 300 K and the NPT ensemble using Berendsen [24] barostatted at 1 atm after a process of 1000 steps of steepest descent energy minimization. Three replicas of 200 ns production MD simulations were performed in an NPT ensemble using a Parinello Rahman [25] barostat. The long-range electrostatic interactions were calculated at every step using the particle-mesh Ewald (PME) [26] method with a cut-off of 1 nm. A cut-off of 1 nm was also applied to Lennard–Jones [27] interactions. All of the analyses were performed using an ensemble trajectory of the last 20 ns of the three replicas. The binding enthalpy between the siRNA and the triblock copolymers was calculated using a molecular mechanics generalized Born model augmented with the solvent-accessible surface area (MM-GBSA) [28]. The stoichiometric value was calculated considering only the PLL segment. The PLL residues were considered complexed only when they were located at a distance of at least 0.3 nm from the siRNA macromolecule.

### 2.8. Estimation of Binding Affinity

ITC measurements were performed on a model VP-ITC titration calorimeter system (MicroCal, Northampton, MA, USA) at 298 K and 1 atm, according to a previous method described by Zheng et al. [29]. Milli-Q water was degassed under vacuum for 10 min and equilibrated to room temperature before use. The titration was initiated after achieving a stable baseline. To prevent any errors due to syringe filling, an initial aliquot of 2 μL was injected. Then, 10 μL of micelles solutions (3 mM nitrogen of PLL_10_; 0.3 mM triblock copolymer; 1.59 mg/mL of polymeric micelles) was titrated to the STAT3-siRNA (0.05 mM phosphate of siRNA; 1.25 μM siRNA; 2 mL) present in the 1.8 mL sample cell. Constant injections, at a spacing time of 180 s, were carried out until a complete saturation of siRNA with the polymeric micelle was obtained. ITC data were analyzed with Origin 7 software (Microcal, Inc., Los Angeles, CA, USA) with a single-site-binding assumption as a fitting model. The association constant (K_a_), dissociation constant (K_d_ = 1/K_a_) and thermodynamic parameters of enthalpy change in binding (ΔH) were calculated by a nonlinear regression. Gibbs free energy (ΔG) and entropy (ΔS) were determined using the following Equations (2) and (3):

ΔG = −RT ln (K_a_), (2)

ΔS = (ΔH − ΔG)/T,(3)

where R is the ideal gas constant (8.314 J K^−1^ mol^−1^) and T is the temperature in Kelvin (K).

## 3. Results

### 3.1. Particle Size, Zeta Potential and Morphology

**Figure 1:**
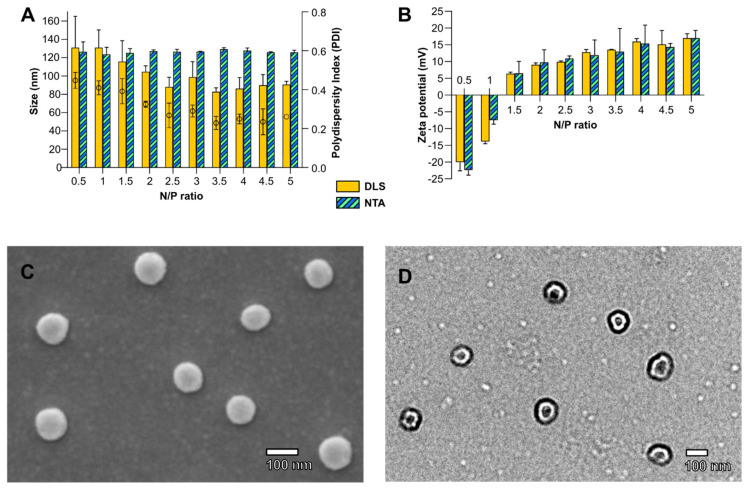
(**A**) The particle size/polydispersity index (PDI) and (**B**) zeta potential of siRNA micelleplexes as determined by the intensity of dynamic/electrophoretic light scattering (yellow-filled bars) and the nanoparticle tracking analysis (hatched bars) at an N/P ratio varying from 0.5 to 5. PDI is represented by ○. Error bars are the standard deviation (SD) of triplicates. Typical images at N/P = 3 by (**C**) SEM and (**D**) TEM.

Micelleplexes were produced at different N/P ratios and characterized using DLS and NTA for the size and the ZP (Figure 1A,B). Morphology and size were analyzed by SEM and TEM, which showed no differences when varying N/P ratios with a typical representation obtained at N/P = 3 (Figure 1C,D). The size and the polydispersity indexes (PDI), measured by DLS, were reduced with the increasing N/P ratio, while the median and mean values remained constant, as shown by NTA. The span values obtained by NTA were also decreased from 0.9 for N/P = 1 to 0.7 for N/P = 5. Negatively charged micelleplexes were obtained at N/P = 0.5 and 1, while positively charged complexes were obtained at 10 mV for N/P = 2 and 15 mV for N/P = 5, as seen by both NTA and DLS techniques. A spherical morphology with a size around 100 nm was confirmed by SEM and TEM microscopy (Figure 1C,D). A neutral charge point around N/P = 1.25 can be observed with a micellar system containing a determined number of cationic charges. The fact that this is very close to the value of 1 shows that the supramolecular arrangement of the polymers reduces the availability of cationic charges, which requires a slightly higher ratio.

A mean of Z-average for all N/P ratios obtained by intensity (DLS) gives a size value around 100 nm (Figure 1A). A median size of 110 nm, based on the number of particles tracked, is observed by NTA at all N/P ratios. The size of micelleplexes is similar for all ratios, as confirmed by microscopy (SEM and TEM). However, the values for each ratio of Z-Ave by DLS, the standard deviations (DLS, NTA), PDI for DLS and span for NTA tend to decrease when increasing the amount of triblock copolymers.

Usually, the average size of the intensity-weighted size distribution is greater than its corresponding numeric weight. Although these values are very close, this seems to contradict the mean sizes obtained by these two techniques, probably due to a different range measurement for NTA. Indeed, NTA studies have reported a practical detection limit of 60 to 70 nm [30].

### 3.2. Complexation Efficacy and Molecular Mechanism of Interaction

**Figure 2:**
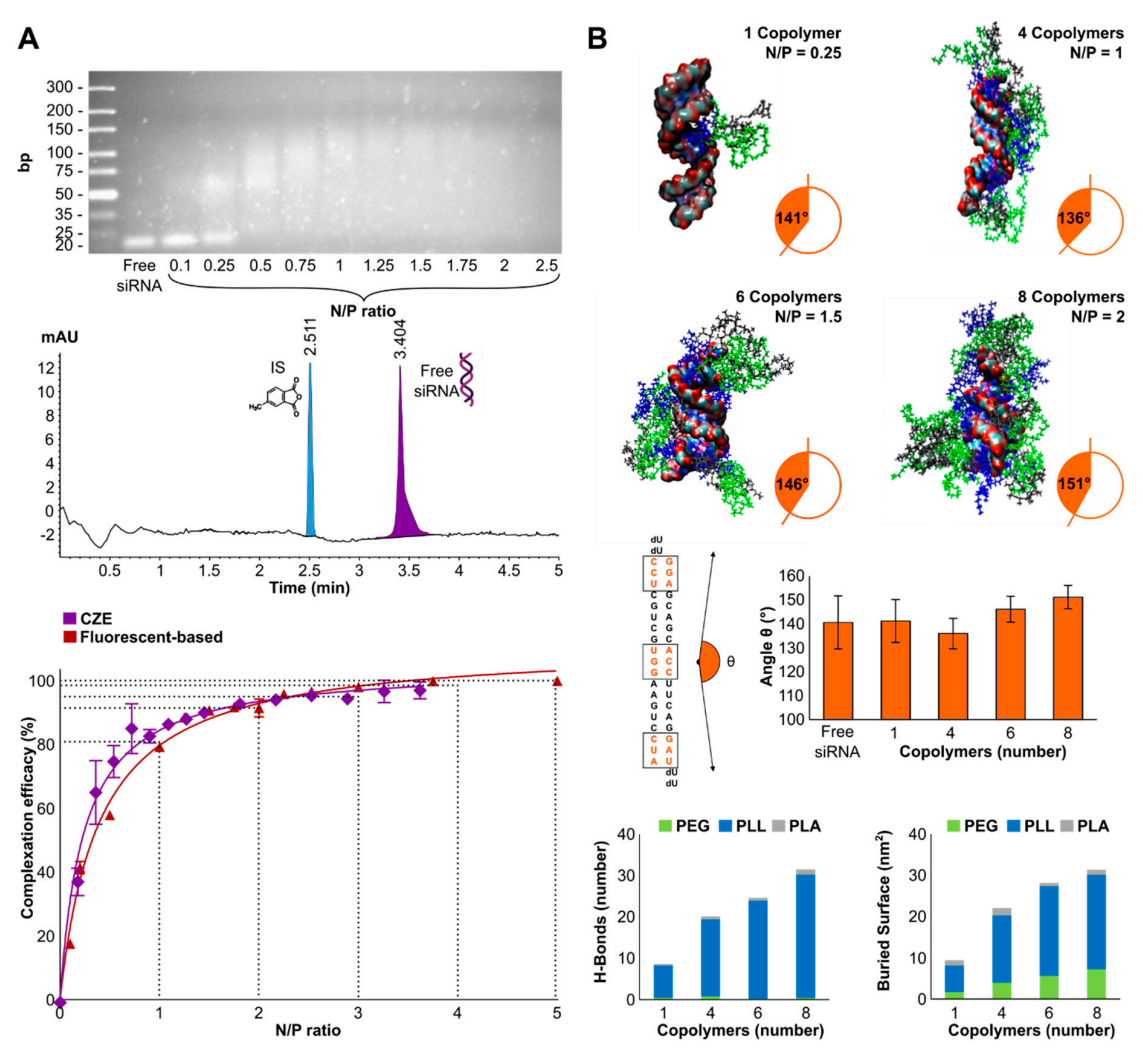
(**A**) Binding properties of triblock copolymeric micelles and STAT3-siRNA run on 4% gel agarose electrophoresis. Typical electropherogram (260 nm) of a standard solution containing 2 μM STAT3-siRNA and 4 μg/mL of the internal standard (IS). Percentage of the complexed siRNA versus the N/P ratio obtained by RiboGreen assay (claret) and capillary electrophoresis (purple) (n = 3). (**B**) Molecular conformation of the complexes by addition of triblock copolymers. Along the filament axis, the angle θ is calculated connecting the nucleotides located at the center of mass and the ending nucleotide bases highlighted in orange. The contribution to the complexation of the polymeric segments PEG, PLL, PLA involved in the complex formation was estimated by calculating the H-bond numbers and the total buried surfaces.

The efficiency of STAT3-siRNA complexation was visualized qualitatively by agarose gel electrophoresis with a complex formation from N/P 0.5 and quantified by CZE and RiboGreen fluorescence-based assay (Figure 2A). The structural conformation of triblock copolymers complexing siRNA was investigated by MM-GBSA. The angle θ of the siRNA, the total buried surface and H-bond number between STAT3-siRNA and the triblock copolymers were estimated computationally (Figure 2B).

A qualitative retention and retardation display can be obtained by gel agarose electrophoresis showing a separation of siRNAs from the polymeric carrier below N/P 0.5 (60% non-complexed siRNAs). The oligonucleotides migrated, as they were not completely retained by the positive charges coming from the carrier. At N/P = 0.25, a smear pattern showing a weak retention linked to an insufficient amount of copolymers showed that the oppositely charged polyelectrolytes were being pulled apart, jointly indicated by Kwok et al. [31]. Above N/P = 0.5, the bound siRNAs were retained in the wells, as observed by smudges with more compact patterns at increasing N/P ratios. Capillary electrophoresis conditions allowed a robust separation, which was demonstrated by the reproducibility of the injection at a standardized concentration of 2 µM of siRNA with an electropherogram presented in Figure 2A. Complexation efficiency can be determined in an automated manner by CZE under simple and inexpensive conditions. Both CZE and Ribogreen techniques showed 80% loading capacity at N/P = 1 and 90% at N/P = 2.

The angle θ analysis (Figure 2B) between the three nucleotides’ center of mass showed that one triblock copolymer branch does not impair the average angle upon interaction. However, the addition of interacting copolymers resulted in a slightly decreased standard deviation of the θ angle with an increased average angle towards a rectilinear geometry of STAT3-siRNA. This shows that the siRNA is stabilized and stiffened when surrounded by the triblock copolymers. Molecular dynamics simulations showed that the PLL segment is the main interacting portion of the triblock copolymer with the siRNA, both in H-bonds (7.8 ± 2.3, 18.7 ± 5.7, 23.7 ± 6.3 and 29.8 ± 3.6 for 1, 4, 6 and 8 copolymers, respectively) and in total buried surfaces (6.5 ± 0.5 nm^2^, 16.3 ± 0.7 nm^2^, 21.8 ± 0.8 nm^2^ and 22.9 ± 1.0 nm^2^ for 1, 4, 6 and 8 copolymers).

### 3.3. Complexation Efficacy and Molecular Mechanism of Interaction

**Figure 3:**
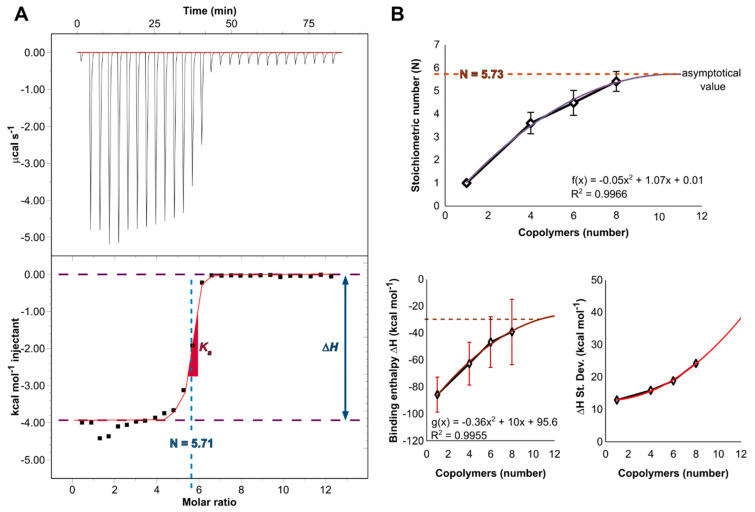
(**A**) ITC thermograph and titration curve-fitting by a one-site binding model (**B**) Interpolation curves by molecular modeling of the stoichiometry, binding enthalpy values and standard deviations following the interaction of one siRNA to multiple copolymers.

In contrast to intensive studies in structure-properties relationship, energetics has received much less attention. Therefore, the differential power (DP) caused by the complexation of the micelles to STAT3-siRNAs was measured and integrated to obtain the binding isotherm (Figure 3A). As such, important information on the interaction of the siRNA with copolymers could be obtained by characterizing in situ their binding thermodynamics. The interaction of one siRNA molecule to multiple copolymeric branches was simulated by molecular modeling (Figure 3B). Asymptotical values of stoichiometry and binding enthalpy energy were extrapolated.

The stoichiometric number of charge (N) was determined experimentally to be (5.71 ± 0.15 copolymers per siRNA). The experimental value of change in free energy (ΔG, Equation (3)) is negative (−42.3 kJ mol^−1^) and confirms a spontaneous phenomenon of non-covalent interactions associated with a strong binding (K_d_ = 38.5 nM). The entropy gain (ΔS > 0, low yet still positive), an enthalpy loss (ΔH = −16.5 kJ mol^−1^), (exothermic; <0) and high values for K_a_ (K_a_ = 2.6 × 10^7^ M^−1^) were obtained. Experimental work has provided a wealth of data for micelleplex formation with attempts to discern the electrostatic contribution from the hydrophobic Van der Waals interaction, hydrogen bonding and hydration force [32]. Due to the complicated processes of the structure formation related to the specific characteristics of two oppositely charged polyelectrolytes, ITC measurements were performed in salt-free water, in order to limit competitive binding. Although, a counterion cloud (CF_3_COO^−^; Na^+^) cannot be completely removed, due to the synthetic procedures, NaCl ions were not added to depict the level of the electrostatic contributions and properly estimate the binding constant. This is consistent with other studies that report that non-covalent interactions are entropy-driven bindings, which proves that the lysine present on the positively charged surface of the micelles interacts primarily with the phosphate backbone of the STAT3-siRNA, which is negatively charged [33], thus suggesting that the H-bonds contribute to the increased stability of these complexes. The inference of the entropic effects from salt dependence has been previously described in the literature [34]. As the incoming copolymers have to displace the salt counterions, this should normally lead to an increase in entropy gain [35].

In parallel, this value was estimated in silico considering only the PLL segment of each copolymer, since they are the predominant interacting subunit of the copolymers, as highlighted in (Figure 2). A rational procedure was followed, considering that the siRNA interacting sites would at a certain point be saturated in the solution, as the oligonucleotides will be completely enshrouded by the triblock copolymers. A promising computational strategy is highlighted here by searching the saturation point of the siRNA sites. This was achieved by extracting the asymptotical values of a polynomial function created by interpolating the stoichiometry data. In this connection, the following polynomial function (f(x) = −0.05x^2^ + 1.07x + 0.01) was used to interpolate the data. The extremum value of the function is located at the saddle point, determined by derivation (f’(x) = 1.07 − 0.1x = 0 ⇔ f(10.7) = 5.73). The average N value of 5.73 was therefore reached at 10.7 copolymers. This value is highly consistent with the experimental one. Regarding the enthalpy profile, a value of −132.2 kJ mol^−1^ reached at 10 copolymers was extracted by the same approach (g(x) = −0.36x^2^ + 10x − 95.6). These data are summarized in Table 1.

Stoichiometry number (N) values were completely consistent. However, inconsistent negative enthalpy values were observed. This seems reasonable and can be explained by different representations of the systems. Indeed, the enthalpic contribution is estimated based on the addition of the copolymeric branches binding to a single siRNA, which is a simplified version of a complete micelle containing several siRNAs used for the experimental measurements.

## 4. Discussion

The characterization methods described in this paper were deliberately limited to easy-to-use techniques. The aim was to provide information on physicochemical characterization based on relatively inexpensive instruments and reduced computational costs (except for electron microscopy). The cheap electrophoresis conditions described in this article could also be implemented using a budget device [36] for quality control of these products in low-income countries, which often struggle to keep pace with technological advances [37].

The use of both DLS and NTA orthogonal sizing techniques is preferable for the robustness of size determination. One can determine size at a broader concentration range and PDI measurements, while the other can give an accurate distribution of size at more diluted concentrations. DLS analysis normally reveals aggregates, whereas herein the more the proportion of copolymeric micelles increases, the more size decreases, showing empty micelles (devoid of siRNAs). This observation does not correlate with NTA, as smaller micelle sizes cannot be detected with this technique; they are shadowed and the system compensates by overestimating micelles of the same size. This limitation by NTA measurement is related to a conversion to a number-weighted distribution.

Among the various size characterization techniques that are available, asymmetric flow field flow fractionation coupled with multiangle light scattering and dynamic light scattering detector (AF4-MALS-DLS) has the potential to become a powerful and very robust method for particle size distribution measurement [38,39]. However, AF4 fractionation requires trained personnel with expertise. This instrument is difficult to use as it is difficult to properly set ideal elution conditions, as shown by the procedure described by Caputo et al. [40], with unsatisfactory measurement of complex structures of the RNA-loaded lipid-based nanoparticles (LNP-RNA) [41]. In a recent article, Mildner et al. explored the use of a frit-inlet channel omitting a focusing step to minimize the contact of LNP-RNA particles with membranes reducing sample loss and particle aggregation [41]. Indeed, sample mass recovery is a key indicator for the evaluation of configuration tests during method optimization and material loss can be more significant for positively charged nanoparticles [41]. Therefore, determining the nucleic acid content per size fraction remains a major challenge. This research is useful, as computational simulations showed convergence with complexation efficacy data, providing conformational elements for a mechanistic description of complex formation.

Regarding complexation efficacy, capillary electrophoresis is a powerful technique that can compete with the commonly used HPLC [42]. Although often referred to as the separation of different oligonucleotides [43], this technique has also been described for the evaluation of the complexation of siRNA to cationic liposomes [12] and by DNA oligomers to polycationic cyclodextrins by hyphenation to mass spectrometry (MS) [44]. Hyphenation of CE to MS opens perspectives by improving the sensitivity within nM range and can also be performed using bare fused silica capillaries for the detection of small RNA oligonucleotides [45,46]. Similar complexation behaviors have been demonstrated using different techniques. In addition, the resulting complexation curves describe a phenomenon of isotherm sorption of STAT3-siRNA, which fits with a Langmuir adsorption model consistent with the uncooperative 1-site model used for ITC. This suggests that adsorbate/adsorbate interactions can be ignored.

The MM-GBSA method faces certain limitations in adapting the ΔH value to the experimental data [16,29,47]. Simulation studies describe molecular conformations, which add further evidence of complexing behavior. Moreover, decreasing ΔH values with increasing copolymers are particularly interesting. By an increase of the standard deviation, due to the increment of copolymers, it is reasonable to hypothesize that the presence of several polycations leads to competition phenomena to bind to the interaction sites. When copolymers are present, (i.e., N/P ratio > 1), competition mechanisms can change certain thermodynamic parameters [17], including the stoichiometric number and the enthalpy, thus explaining a visible decrease in ΔH. These competition phenomena suggest a greater instability of the system and a challenge to maintaining a point of charge-neutralization, which may ultimately hinder efficient cell transfection.

## 5. Conclusions

In this study, we presented cross-cutting techniques that bridge experimental and computational methods to gain knowledge of how electrostatic interaction will affect complexation behavior and stability. This interdisciplinary research area helps ensure an optimized physicochemical characterization of siRNA-loaded micelleplexes. With respect to translational research, this approach might help limit the number of cell-based assays. An alternative approach by combinatorial library of polymers is suggested to be especially consuming. Although this work is limited to characterization without a direct relationship to biological fate, the approach could be further considered for a better design of novel cationic polymers. Further studies are needed to shed light on toxicity and efficacy implications. The thermodynamics of interaction help understand the molecular basis.

We have used different techniques to characterize the binding formation, which has significant advantages as compared to the classical gel retardation assay. Moreover, an N/P ratio of 1.5 should be selected to provide a high complexation efficacy of 85%, resulting in slight positively charged micelleplexes. The ratio of N/P = 1.5, slightly higher than the theoretical value of N/P = 1, was demonstrated in connection with the molecular orientation of the triblock copolymers complexed around the double-stranded siRNA. By describing a variety of easy-to-implement methods, we showed how to determine an appropriate range of N/P ratios. Suitable and reliable analytical techniques were offered to screen important physicochemical parameters such as size, ZP, morphology, which may be regarded as critical quality attributes (CQAs). In the future, an important feature should be developed to correct drift and limit discrepancies in enthalpy values. We recommend normalizing values against a calibration material.

## Figures and Tables

**Table 1:** Summary of the energetics parameters involved in the molecular interactions.

	N (sites)	K_a_ (M^−1^)	K_d_ (nM)	ΔH (kJ mol^−1^)	ΔS (kJ K^−1^ mol^−1^)	ΔG (kJ mol^−1^)
Experimental	5.71 ± 0.15	2.6 · 10^7^	38.5	−16.5	0.087	−42.3
Computational	5.73	N/A	N/A	−132.2	N/A	N/A

## Data Availability

Not applicable.
